# Feasibility study for the automatic surgical planning method based on statistical model

**DOI:** 10.1186/s13018-023-03870-x

**Published:** 2023-06-01

**Authors:** Hang Phuong Nguyen, Hyun-Joo Lee, Sungmin Kim

**Affiliations:** 1 Department of Electrical, Electronic, and Computer Engineering, University of Ulsan, Ulsan, Korea; 2 Department of Orthopaedic Surgery, School of Medicine, Kyungpook National University, Kyungpook National University Hospital, Daegu, Korea

**Keywords:** Template, Statistical shape model, Computer-assisted surgical planning, Clinical decision support

## Abstract

**Purpose:**

In this study, we proposed establishing an automatic computer-assisted surgical planning approach based on average population models.

**Methods:**

We built the average population models from humerus datasets using the Advanced Normalization Toolkits (ANTs) and Shapeworks. Experiments include (1) evaluation of the average population models before surgical planning and (2) validation of the average population models in the context of predicting clinical landmarks on the humerus from the new dataset that was not involved in the process of building the average population model. The evaluation experiment consists of explained variation and distance model. The validation experiment calculated the root-mean-square error (RMSE) between the expert-determined clinical ground truths and the landmarks transferred from the average population model to the new dataset. The evaluation results and validation results when using the templates built from ANTs were compared to when using the mean shape generated from Shapeworks.

**Results:**

The average population models predicted clinical locations on the new dataset with acceptable errors when compared to the ground truth determined by an expert. However, the templates built from ANTs present better accuracy in landmark prediction when compared to the mean shape built from the Shapeworks.

**Conclusion:**

The average population model could be utilized to assist anatomical landmarks checking automatically and following surgical decisions for new patients who are not involved in the dataset used to generate the average population model.

## Introduction

Computer-assisted surgical planning is a preoperative procedure that consists of significant tasks like surgical target identification, surgical access planning, surgical tools and implant positioning, and assessment of the selected plan [1]. It supports surgeons effectively in making clinical decisions and improving outcomes in many kinds of surgery like orthopedic surgery [2], cranio-maxillofacial surgery [3, 4], neurosurgery [5–7] . Particularly, there was previous work using computer-assisted surgical planning for reverse shoulder replacement [8] and spine [9]. Moreover, researching interpatient variability for surgical planning is essential because the dramatical difference between individuals in the inherent and morphometrics (or shape analysis) of anatomical structure would affect the success of a surgery, for instance, in orthopedic implants [10–12], craniosynostosis surgery [13], and head-and-neck cancer resection [14]. Therefore, studies based on anatomical shape have evolved into an indispensable part of surgical planning. In this study, we built average population models from the right and left humerus bones of female and male cadavers to share an idea of how to exploit an anatomical population-based model for the computer-assisted surgical planning, and the aim of our study is to reveal the usability of the anatomical population-based model for exact surgical landmark checking and surgical decision making. Results of this paper could be considered as a positive approach for making decision of humerus surgery scenarios.

### Related work

Studies in anatomical shape analysis started more than 100 years ago, and statistical shape modeling (SSM) has become a great change in morphometric techniques to visualize complex anatomical structures and their variability in the population at a precision level by using statistical power [15–17]. SSM provides two kinds of information about the population: (1) *the mean shape*—an average shape over all involved shapes in the population; (2) *the variation parameter* to represent how much the shape can differ between subjects in the population [18]. Quantitative results of SSM assign a normal anatomical structure with a high probability, while assign a low probability for a pathological shape [19]; therefore, SSM has been applied widely to computer-assisted surgical planning, including radiotherapy planning [20], orthopedic surgery [21], spring-assisted cranioplasty [22], and cervical adaptive radiotherapy [23]. In the field studies of SSM, a well-defined correspondence technique is a prerequisite for building a statistical model, and computing correspondences automatically is based on registration between involved shapes [18]. Based on the review of *Oguz *et al. about correspondence techniques [24] and the evaluation and validation of *Goparaju *et al. about statistical shape modeling tools [16, 25], we can classify approaches for defining correspondences into two categories: the groupwise method and pairwise method.

The groupwise method mostly evolved from the pioneer model of point distribution models (PDM) [26] which considered representing objects or images as a set of points, and using principal component analysis (PCA) to build the statistical model. Many studies have developed from the theory of PDM using open-source or SSM tools for analyzing general anatomies, for example, minimum description length (MDL) [27], Statismo framework [28], and Shapeworks [15]. The study of *Davies *et al. [27] establishes optimal correspondence automatically between sets of shapes by applying the principle of MDL, whereas Statismo [28] implements probabilistic PCA to interpret the modeled objects, and statistical models generated by Statismo are represented as a probability distribution. The Shapeworks [15] proposed Particle-based modeling (PBM) where point-to-point correspondences between involved shapes are represented as dynamic particles which freely move on the surface of the modeled shapes and the positions of the particles can be directly optimized. The highly significant contribution of the Shapeworks to correspondence optimization is the algorithm of entropy minimization in shape space, and the effectiveness of the Shapeworks has been demonstrated in a range of medical and clinical applications including orthopedics, cardiology, hip joint FAI pathology, dysplastic hip joint, scapular morphology in Hill–Sachs patients, atrial fibrillation, and so on [15, 25]. Even though the Shapeworks showed the effectiveness and potential ability for applying to clinical applications, the principle of the Shapeworks is still built on the idea that there are point correspondences between involved structures, and almost modeled objects using Shapeworks are bones with a relatively stable shape over the population. To deal with complex structures like cardiac and vessel systems, or deal with highly varying soft tissues like liver and surfaces segmented MR images, point-based approach is difficult to establish point correspondences and to generate a statistical model [18, 29, 30].

The pairwise method, on the other hand, establishes the correspondences by mapping each involved subject to a predefined atlas or template following the principle of surface-based pairwise or volume-based pairwise correspondence [24]. The approach of surface-based pairwise requires a standard parameter space where each object is mapped to, and the correspondences are computed between the individual samples and the parameter space. Most of surface-based pairwise approaches used a sphere as the standard space; for instance, *Kelemen *et al. proposed a spherical harmonics (SPHARM) [31], and then, *Styner *et al. developed a SPHARM-PDM framework for building statistical shape analysis of brain structure [32] or hippocampus in schizophrenia [33]. However, the evaluation of *Goparaju *et al. [25] pointed that SPHARM-PDM displayed inferior results compared to Shapeworks in the evaluation and validation experiments for clinical applications. Another approach of the surface-based pairwise is using a nonparametric representation of shape as current a mathematical object to characterize geometrical data via vector field—and the correspondences are computed in the space of currents based on rigid registration. Nevertheless, the current-based approach mainly depends on some parameters which used to model the geometrical data, such as the spatial scale of the currents and the scale of deformation [29, 30]. On the other hand, the volume-based pairwise method is based on a principle of not existing explicit correspondences between the individuals, and researches for shape analysis using the volume-based pairwise recently have shown positive results for the human brain [34, [35], cardiac [30], heart [36], and even brain template of non-human macaque [37]. However, these studies focused on analyzing shape models of soft tissues which have dynamic shape, not stable shapes like bones. Between some studies which focused on the volume-based pairwise such as FNIRT [38] and DRAMMS [39], an open source named Advanced Normalization toolkit (ANT) showed precision results in building template with high accuracy in registration when compared to others open sources [40, 41]. Processing for creating an ANTs template does not bias toward any individuals, and the template generated from ANTs represents an unbiased average of involved shapes in the population [34].

### Contributions

In this study, we aimed to establish computer-assisted surgical planning method based on human population data set. For that, the ANTs were applied to build average population models from inter-humerus datasets that include males and females with full corresponding left and right bones. Based on the average population models, the automated computer-assisted surgical planning method could be established, the surgical planning was conducted on the average population model, and the planning data could be transferred to each individual data even the data are not involved the dataset which is used to build the average population model.

To support the main idea for the computer-assisted surgical planning method, evaluation and validation experiments were conducted to make surgical predictions for new data sets that did not involve in the procedure of building average population model. Mean shapes generated from Shapeworks were used as references to compare evaluation results and validation results to the ANTs templates.

## Materials and methods

**Fig. 1:**
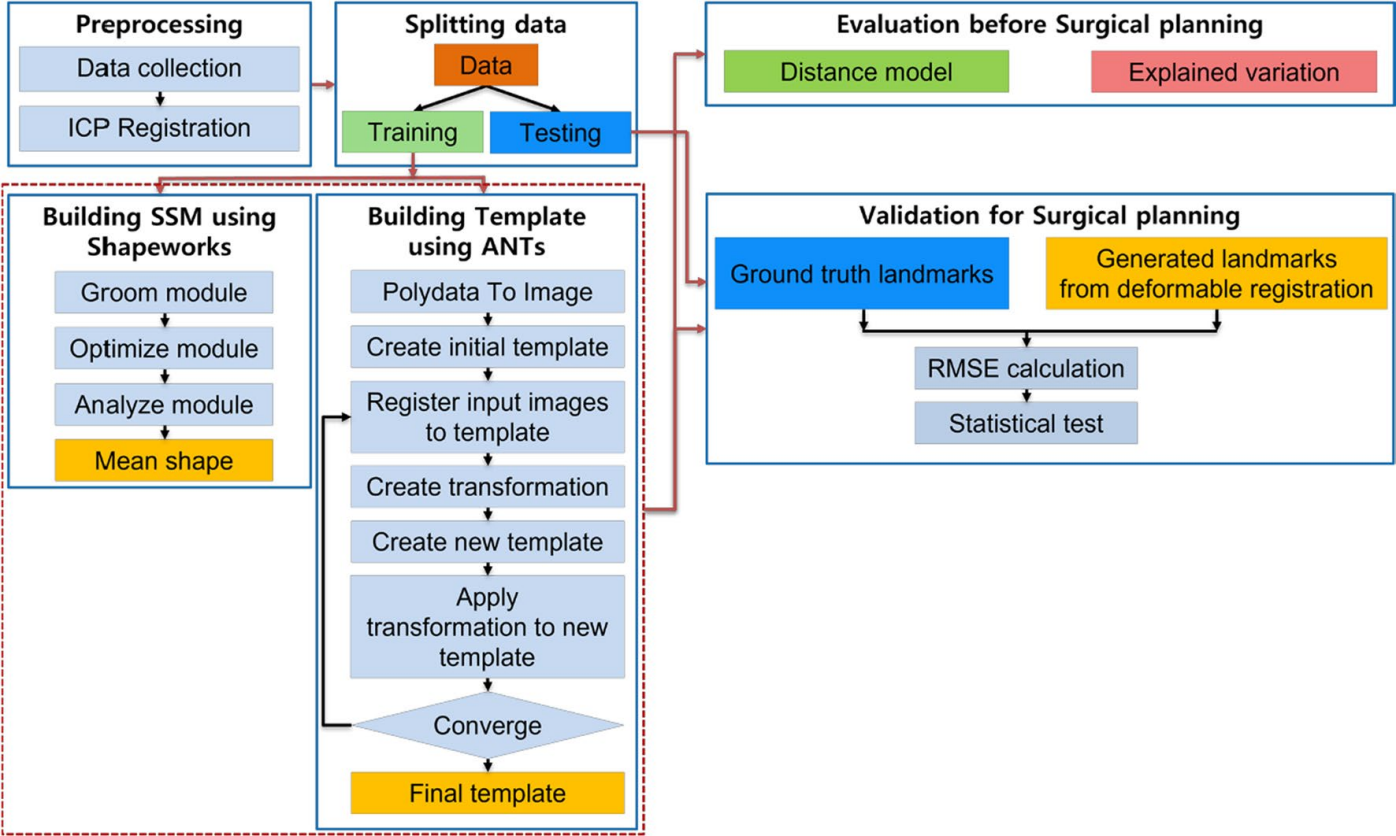
Framework for using the average population model in the surgical planning

The framework for building average population model and applying it to the surgical planning is presented in Fig. 1. The steps in the framework consist of (1) preprocessing; (2) splitting data; (3) building the average population model; (4) evaluating the average population model before using it in the surgical planning; and (5) validation for the surgical planning.

### Preprocessing and splitting data

The preprocessing consists of collecting data and data alignment based on iterative closest point (ICP) registration.

The data used for evaluation and validation are polydata of humerus bones that were collected from a database of the Korea Institute of Science and Technology Information (KISTI). The dataset of humerus includes 50 female subjects and 43 male subjects with full left bones and right bones. We separate into four sub-datasets that consist of female-left, female-right, male-left, and male-right sub-dataset. The dataset is written on the file with STL file format.

In the data alignment stage, with each sub-dataset, we select the first humerus subject as an initial reference and perform the ICP registration between the reference and each of the remaining subjects in the sub-dataset. The alignment process is implemented using an extension that our group built on the 3D Slicer [42].

To form data for training and testing, we randomly select subjects with a ratio of 82% for training and 18% for testing without bias in each sub-dataset. The average population models are built using Shapeworks and ANTs on the training data. The testing data sets are used for validation in the context of surgical planning.

### Building average population model

We use the Shapeworks to build the SSMs and apply the ANTs to generate the templates. Shapeworks provides a convenience all-in-one GUI-based interface called Shapeworks Studio to build SSM, which includes the groom module, optimizes module, and analyzes module, as shown in Fig. 1. The humerus dataset was preprocessed and registered as described in Sect. 2.1; therefore, we chose the option of skipping grooming in the groom module. The optimized module provides options to model correspondence dynamic particles between the individuals using entropy minimization [15]. After the optimization process is completed, a mean shape of polydata is extracted from the analyze module.

A template of ANTs is a population-average image that is unbiased with respect to both shape and appearance from individuals [34], and the process to build the template is presented in Fig. 1. First, to adapt to ANTs’ data format, we convert original polydata in each sub-dataset into images using Visualization Toolkit (VTK) [43]. The subject image with the biggest volume is chosen as an reference coordinate to define the space for an initial template, and each subject image is resampled with respect to the initial template. The intensity of the initial template is computed as voxel-wise average from training images. An iterative nonlinear registration process is applied to build the population-average template as follows:

- Each image is registered to the temporary template using affine and deformable symmetric normalization (SyN) transformation [34, 44].
- The inverse transformations from the temporary template to each of the subject images are averaged to create a new transformation.
- The registered images are averaged to update the temporary template.
- New transformation is applied to the updated template.
- The process is iterated, and it will be completed if the difference between updated templates is minimized. The empirical research shows that four iterations are sufficient for building an optimal template in ANTs.

### Evaluation before surgical planning

**Fig. 2:**
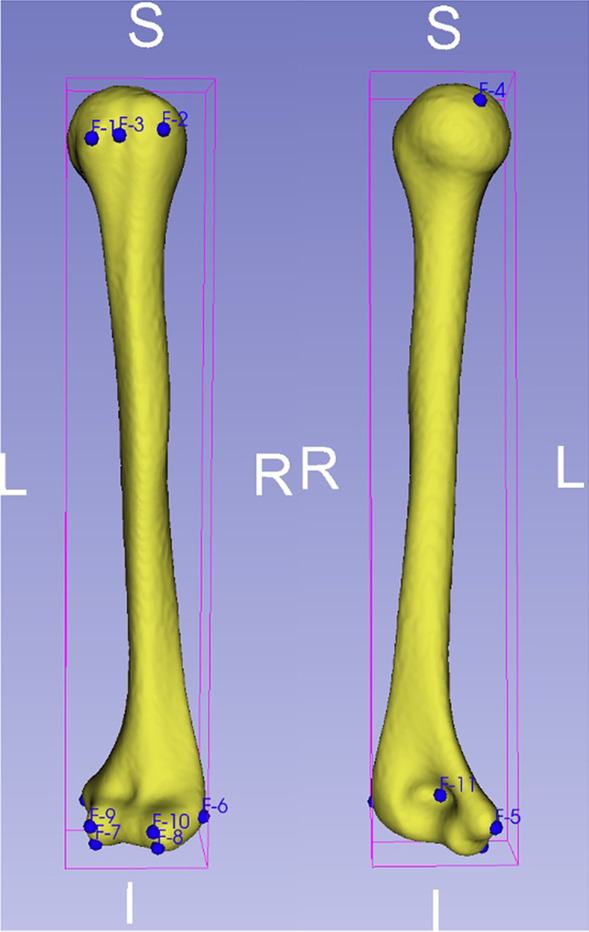
Eleven clinical landmarks are defined on the humerus

Before using the average population models for surgical planning, explained variations are calculated to evaluate how much variance of shape of individuals can be explained by the average population model. The explained variations of mean shape are extracted from the analyze module of the Shapeworks Studio. For the template of ANTs, the explained variations are calculated using a principal component analysis (PCA). First, a group of 11 anatomical landmark points which are widely used for clinical communication and as surgical landmarks are determined on the template by an orthopedic surgeon, as represented in Fig. 2. Next, these landmarks from the template (fixed image) are transformed to each subject image (moving image) in the training data using deformable registration to generate the correspondence landmark point clouds. After applying PCA, the percentage of explained variation in each mode (principal component) is computed using its eigenvalue divided by the sum of all the eigenvalues [36]. Note that the number of modes is equal to the number of individuals minus one [28].

To evaluate the shape of the average population model, distance models and distance histograms between the mean shapes and the ANTs templates are computed in 3D Slicer. The ANTs template is reconstructed into a polydata using segmentation module of the 3D Slicer [42] before registered to the mean shape using ICP registration. Next, a Hausdorff algorithm from VTK is applied to compute the distance model.

### Validation for surgical planning

The validation for the context of surgical planning is performed as follows:

- Eleven landmarks are manually determined on the average population model and each subject in the testing data by an expert at those clinical positions where presented in Fig. 2. The landmark annotations of subjects in testing data work as subject-specific ground truth.
- Eleven landmarks on the average population model are transferred to the testing subject using affine and deformable B-spline SyN [45]. The transferred landmarks work as subject-predicted landmarks.
- Calculate RMSE between the ground truth and the predicted landmarks.
- Apply paired t tests.

## Results

**Fig. 3:**
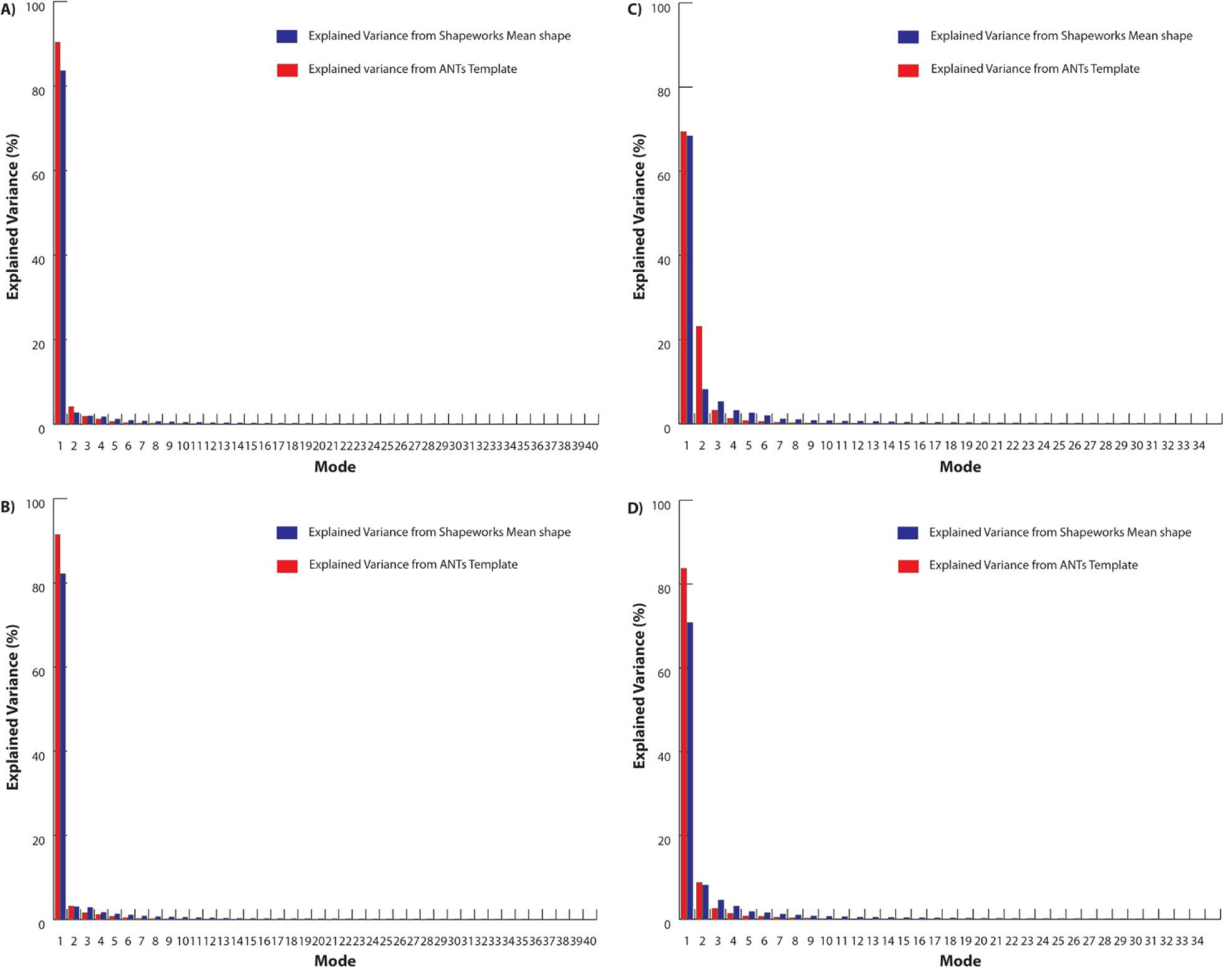
The explained variation of the average population models in each sub-dataset: **a** female-left, **b** female-right, **c** male-left, **d** male-right

The explained variation for each sub-dataset in cases of modeling by Shapeworks and ANTs is presented with graphs (Fig. 3). Figure 3 shows that in all cases of sub-dataset, the first seven modes of the average population model can capture 99% the shape variation of the individuals. However, the templates generated by ANTs can capture the variance of shape across individual subjects with higher explained variation than the mean shapes of Shapeworks.

**Fig. 4:**
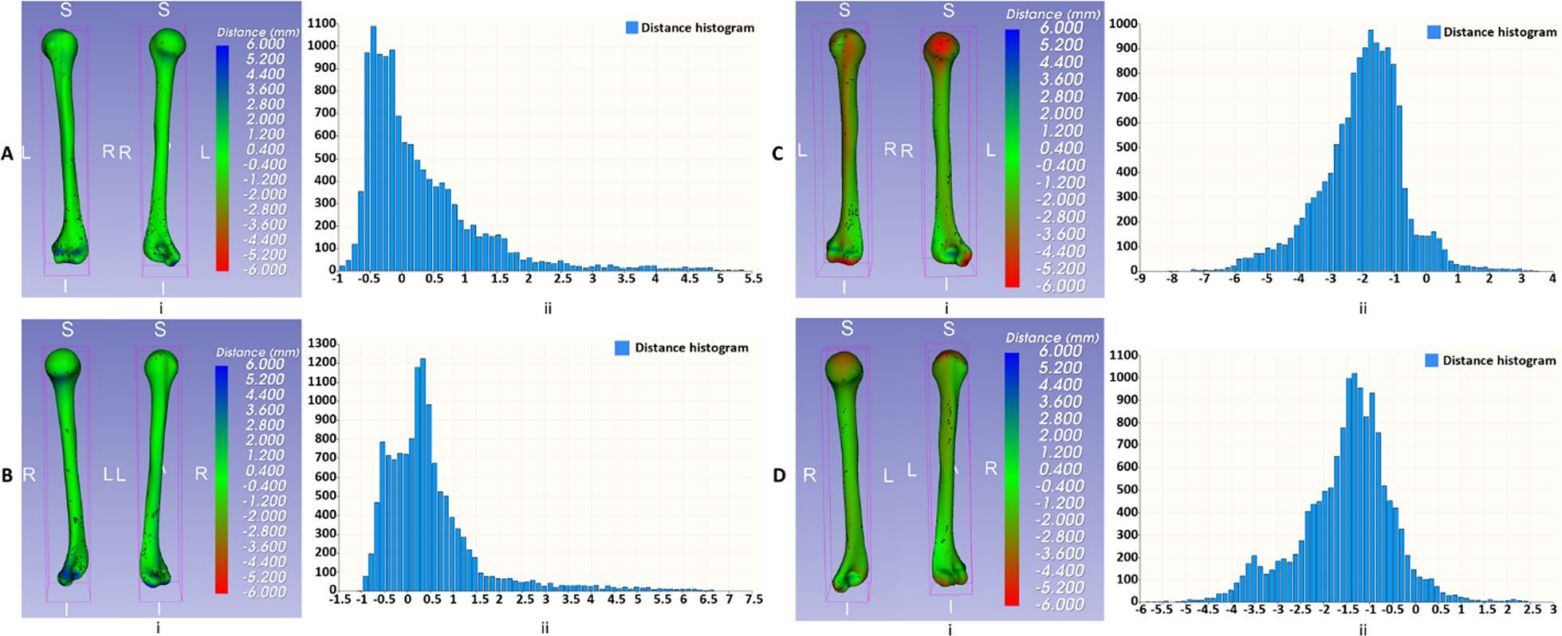
Distance models and correspondence histograms of distance values for each sub-dataset: **A** female-left, **B** female-right, **C** male-left, **D** male-right

Figure 4 shows an evaluation of the shape of the average population models as distance comparisons between the template and the mean shape. A RGB color map was presented to encode the distance range from − 6 to 6 mm, and a signed distance means that one model is inside the other.

First, we consider the distance model and the histogram in the case of female subjects as shown in Fig. 4A, B. The distance models of female-left and female-right display primarily in green color, and the highest frequency bins of the histogram represent distance values in a range from − 0.5 to 1 mm. These results show that there are no significant differences in shape between the ANTs template and the mean shape in two cases of female-left and female-right.

Second, in the case of male subjects, the highest frequency bins of the histogram represent distance values in a range from − 2.5 to − 1 mm in case of male-left, and a range from − 2 to 0.5 mm in case of male-right. There are some small red regions on the head of the distance model of male-left and male-right but insufficient to affect the global shape of the ANTs template and the Shapeworks mean shape.

**Fig. 5:**
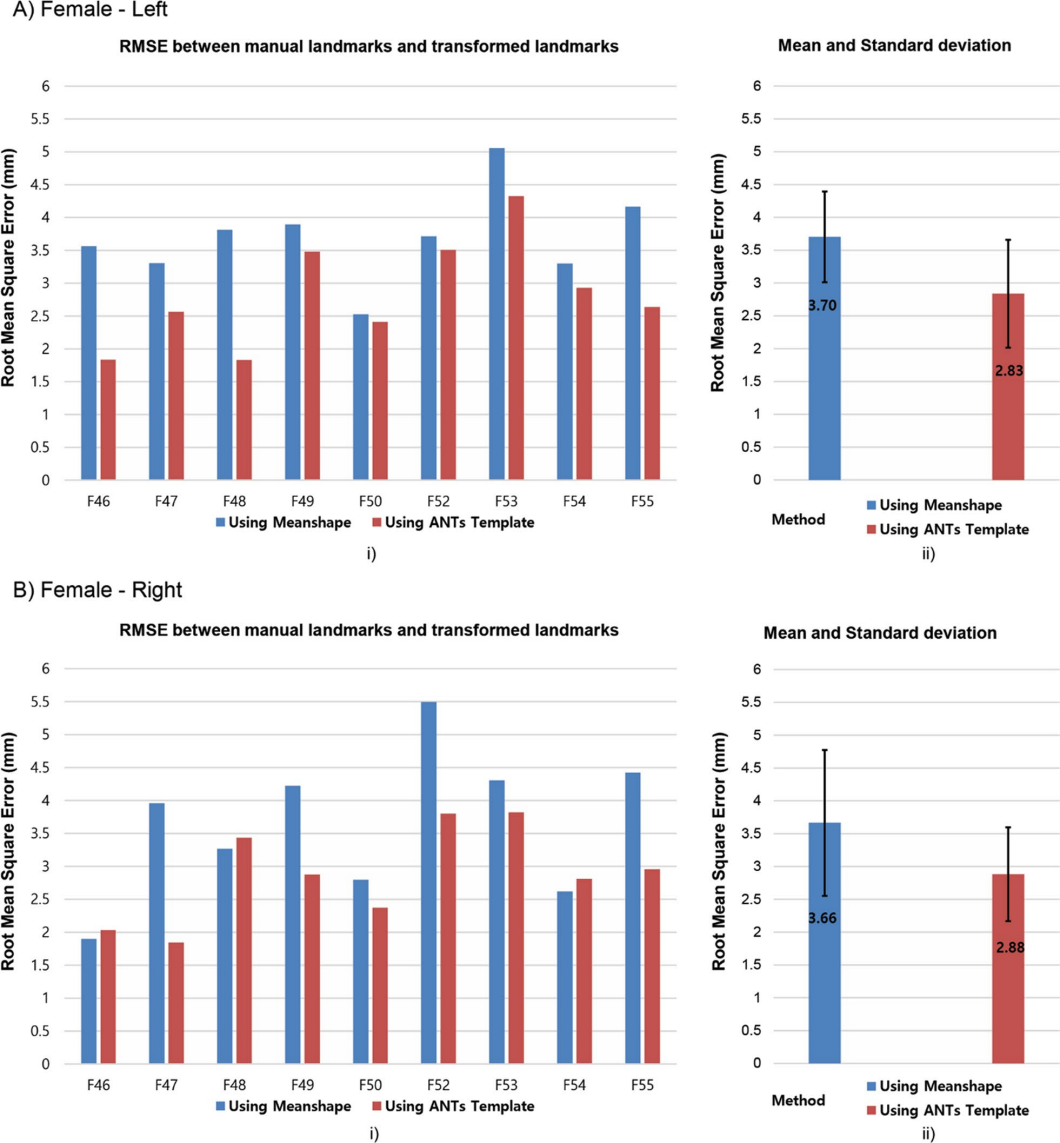
Humerus landmarks differences in ground truth and prediction using average population models for female: **A** left (p value < 0.05), **B** right (p value < 0.05)

**Fig. 6:**
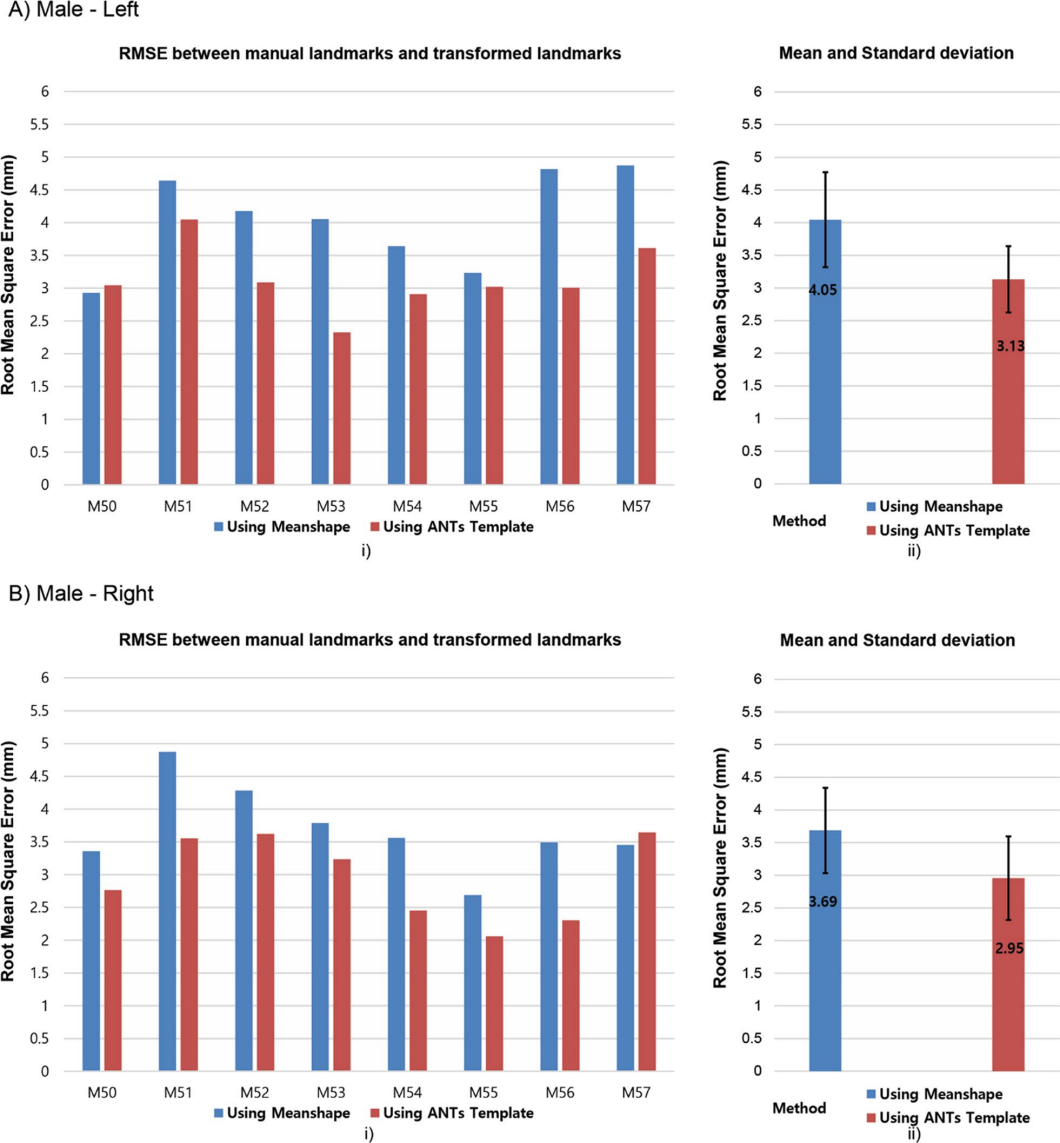
Humerus landmark differences in ground truth and prediction using average population models for male: **A** left (p value < 0.05), **B** right (p value < 0.05)

Figures 5 and 6 present the differences in clinical landmark’s position between the ground truth defined by the expert and the predictions generated by the average population models. When using ANTs template, the minimum average of RMSE is 2.83 mm and the maximum average of RMSE is 3.13 mm, while the minimum average and the maximum average of RMSE in case of the Shapeworks mean shape are 3.66 mm and 4.05 mm, respectively.

## Discussion and conclusion

In clinical setting, surgical landmarking is crucial for surgical planning. The precise landmark is utilized to measure the bone parameters including its length and width. To insert the implant properly, accurate landmarking is the first step for surgical planning. Moreover, proper specification of the landmark is essential for the development of bone implants, such as bone plates or total joint arthroplasty. We built the average models for automated computer-assisted landmarking, aiming to improve surgical planning. In this study, we evaluated the feasibility of using the ANTs template compared to the mean shape of the Shapework for surgical planning. The experiments include evaluations of the shape of the average population model and validation of predicting surgical landmarks positions for new data using the average population model.

The shape of the average population model is a primary factor that needs to evaluate before making decisions for surgical planning. Results from Figs. 3 and 4 show that the ANTs template works as a good average population model for modeling the shape variation of individuals in the training dataset with higher explained variance when compared to the Shapeworks. The higher the explained variance of the average population model, the more the model can explain the variation of the shape of the individuals in the data.

Figures 5 and 6 present that the average population model built from the Shapeworks or ANTs could use to make predictions for clinical landmarks locations with acceptable errors for new humerus data that were not involved to the process of building the average population model. However, ANTs templates presented an improvement in landmark prediction when compared to the mean shapes built from Shapeworks because using the ANTs template provides improved accuracy with approximately 23%, 21%, 22%, and 20% in cases of female-left, female-right, male-left, and male-right, respectively. Despite the average models generated from small-size datasets, the ANTs templates showed highly efficient results when transferred the surgical landmarks closely to the ground truth. These precision results are explained by using SyN algorithm in ANTs. The SyN algorithm showed the most consistently high accuracy registration across subjects in an evaluation of 14 nonlinear deformation algorithms [41].

In this study, we shared the idea that we could use the average population model to support surgical decisions automatically for new patients who are not involved in the dataset building the average population model. Our results could be considered for designing an automated computer-assisted surgical planning method using ANTs.

In the future, we plan to extend the dataset to build more robust average population models and conduct the experiments applying the average population model to real surgical planning with the humerus or other anatomical structures.

## Data Availability

The data that have been used are confidential.

## Abbreviations

ANTs: Advanced Normalization Toolkits
RMSE: Root-mean-square error
SSM: Statistical shape modeling
PDM: Point distribution models
PCA: Principal component analysis
MDL: Minimum description length
PBM: Particle-based modeling
SPHARM: Spherical harmonics
ICP: Iterative closest point
KISTI: Korea Institute of Science and Technology Information
VTK: Visualization Toolkit
SyN: Symmetric normalization
PCA: Principal component analysis
